# The translation and validation of the Arabic Version of the Polycystic Ovary Syndrome Health-Related Quality of Life Questionnaire (AR-PCOSQ)

**DOI:** 10.1186/s12905-020-01108-0

**Published:** 2020-10-29

**Authors:** Sultan Alghadeer, Alhanouf Algarawi, Faten Abu-Rkybah, Mashael M. Alshebly, Yazed Alruthia

**Affiliations:** 1 Basic Sciences Department, Prince Sultan College for Emergency Medical Services, King Saud University, Riyadh, Saudi Arabia; 2 Department of Clinical Pharmacy, College of Pharmacy, King Saud University, P. O. Box 2457, Riyadh, 11451 Saudi Arabia; 3 Obstetrics and Gynecology Consultant, King Saud University Medical City, Riyadh, Saudi Arabia; 4 Department of Obstetrics and Gynecology, College of Medicine, King Saud University, P. O. Box 2925, Riyadh, 11461 Saudi Arabia

**Keywords:** Polycystic ovarian syndrome, Arabic, Questionnaire, Translation, Validity, Reliability

## Abstract

**Background:**

Polycystic ovarian syndrome (PCOS) is a hormonal disorder that is prevalent in females of reproductive age with signs and symptoms that significantly reduce self-esteem and have a negative impact on their quality of life. The management of PCOS signs and symptoms should result in an improvement in the health-related quality of life (HRQoL) of patients. Polycystic ovarian syndrome questionnaire (PCOSQ) is a disease-specific scale. The PCOSQ has been translated into different languages and assessed in different populations. The validity and reliability of PCOSQ varied depending on the ethnicity and culture of the respondents. The objective of the study was to establish a valid and reliable version of the PCOSQ (AR-PCOSQ) in Arabic.

**Methods:**

A cross-sectional study using the translated and validated AR-PCOSQ questionnaire was conducted by interviewing 117 women with PCOS.

**Results:**

The mean age (years) and BMI (kg/m^2^) of subjects were 29.90 ± 6.33 and 27.21 ± 5.54, respectively. Most of the patients had ≥ 1-year long history of PCOS (73.5%) and a post-school degree (64.96%). The content validity index (CVI) for the AR-PCOSQ from 10 gynecologists was 0.9, indicating satisfactory validity content. The internal consistency for reliability confirmation measured by Cronbach’s alpha coefficient was applied. Alpha coefficients for all items together was 0.863, indicating good reliability. The intraclass correlation coefficients for each item for 30 participants were also acceptable, ranging from 0.911 to 0.986 with *p* value < 0.001. As far as the factor analysis is concerned, the overall Kaiser–Meyer–Olkin sampling adequacy measure was 0.772. The Bartlett sphericity test was significant (*p* ≤ 0.001), Indicating that there were interrelated variables.

**Conclusion:**

Our results demonstrated the initial reliability and validity of the Arabic version of the PCOSQ as a measure of specific HRQoL in Saudi women with PCOS. This will fill an important gap in measuring the HRQoL for patients with PCOS in research and community settings in Saudi Arabia. The AR-PCOSQ can be used to help prioritize health-related concerns from the patient’s perspective.

## Background

One of the prevalent endocrine disorders among women of reproductive age is polycystic ovarian syndrome (PCOS). The global prevalence of PCOS is estimated to range between 5 and 10%; however, its prevalence in Saudi Arabia is largely unknown [1, 2]. This syndrome includes a myriad of signs and symptoms such as amenorrhea or oligomenorrhea, obesity, infertility, anovulation, acne, and hirsutism [3, 4]. These signs and symptoms can significantly lower patients’ self-esteem and negatively impact their physical quality of life [4, 5]. Furthermore, PCOS negatively affects the mental well-being of the affected patients resulting in both depression and anxiety [6]. Therefore, managing PCOS signs and symptoms should result in an improvement in patients’ health-related quality of life (HRQoL) [7].

Measuring HRQoL for PCOS patients at baseline as well as after the impact of different treatment approaches is informative and helpful to clinicians and patients alike to determine the value of each treatment approach from the patients’ perspective [8].

Today, many clinicians, health care policy makers, and regulators are interested in Patient-Reported Outcomes (PROs) to assess the quality of provided care. Of those PROs, HRQoL is considered one of most important measures in assessing the quality of care. Therefore, to evaluate the HRQoL among different patient populations, several scales have been developed These scales can be generic (whereby they are used to assess the HRQoL among patients with different medical conditions) or disease-specific [9]. Generic HRQoL scales such as the Short Form Health Survey (SF-36) or abbreviated version of World Health Organization Quality of Life (WHOQOL-BREF) scale may not be as accurate as the disease-specific HRQoL scales in measuring the HRQoL among patients with specific health care conditions such as PCOS [10]. PCOS-specific HRQoL scales such as the polycystic ovarian syndrome questionnaire (PCOSQ) have been developed to address different domains in patient HRQoL relevant to PCOS, since there is no specific HRQoL instrument to use in women with PCOS.

Despite its specificity, different HRQoL outcomes among women with PCOS from different backgrounds confirm that in women with PCOS, ethnicity and culture play an important role in evaluating the quality of life. Chinese, Korean, South African, and Swedish versions of the PCOSQ were found to be reliable, valid, and culturally acceptable [11–14]. In the UK, PCOSQ was found to be reliable, but its validity needs to be improved by incorporating a dimension on acne [15]. In Iran, except for menstrual issues, the questionnaire was found to be accurate and valid in all aspects [16, 17].

The PCOSQ could be used to identify issues associated with the HRQoL in women with PCOS, evaluate the full effectiveness of treatment regimes, identify and report changes in patients' health status over time, and generate more understanding of the impact that the symptoms and treatments of PCOS could have against HRQoL. However, a variation in validity and reliability of PCOSQ was noticed among women from different ethnicities and cultures. The aim of this study was to establish a valid and reliable Arabic version of the PCOSQ.

## Methods

### Study design and participants

A cross-sectional study using the translated questionnaire (AR-PCOSQ) was conducted by interviewing 117 PCOS women who attended King Khalid University Hospital (KKUH) obstetrics and gynecology clinics during the period from November to December 2017. The institutional review board (IRB) at KKUH granted ethical approval (IRB Project No. E-17-2545). The criteria for inclusion were 18–45 years of age, married, Arabic-speaking women with no verbal communication difficulties. Non-classic adrenal hyperplasia, thyroid dysfunction, hyperprolactinemia, previously diagnosed diabetes, any drug with an effect on insulin levels, or hormonal drugs (including contraceptive pills) were excluded at least two months before participating.

### Measurement

A specific tool to evaluate the HRQoL of women with PCOS is PCOSQ. It is composed of 26 items categorized into five sections: emotions (eight items), body hair (five items), weight concerns (five items), infertility concerns (four items), and menstrual irregularities (four items). Each item is scored with a seven-point Likert rating system where seven represents the best situation and 1 represents the worst situation. The emotions section asks questions related to mental illness, depression, mood, self-esteem, and fear of cancer as consequence of PCOS diagnosis. The body hair section contains items concerning noticeable hair in face and overabundance of hair throughout the body. The issues of gaining weight or being overweight and infertility issues such as inability to conceive and having children are included in the weight and infertility concerns sections, respectively. The menstrual irregularities section includes items regarding abnormalities in menstrual periods and body discomforts secondary to menstrual abnormalities. The mean score for all items in a section indicates the score for each woman [18].

### Procedure

Two interviews were conducted to assess the face validity and test–retest reliability. The first interview was face-to-face interview while the second interview was conducted subsequently via the phone during an interval period of 5 days to 2 weeks post the face-to-face interview. This interval was selected in order to maintain the consistency and similarity of PCOS condition as the quality of life changes when PCOS condition changes with time progress.

Use of the Polycystic Ovary Syndrome Questionnaire, authored by Dr. Gordon Guyatt, et al. was made under license from University, Hamilton, Canada, with the permission of the copyright owner, The Endocrine Society, Maryland, USA. A forward–backward process has been used to translate the English version of the PCOSQ into Arabic. Two bilingual translators (one with a medical experience and one without) proficiently fluent in English each independently translated the complete English version of the PCOSQ into Arabic, including item content, response options, and instructions. The two forward translations were merged into one version by primary investigators after the expert panel discussions that consist of gynecologists, professional translators, and experts in public health. Any discrepancies between the forward translations will be identified and resolved by the expert panel. Two translators independently later translated the single forward translation back into English in the backward translation while totally blinded to the PCOSQ's original English version.

### Statistical analysis

Using IBM SPSS Statistics, statistical analysis was carried out. To test the validity of the Arabic translated version of PCOSQ, face and content validity was used. Reliability analysis of reliability of the test–retest as well as internal consistency were carried out.

### Content validity

The content validity index (CVI) of the items was calculated based on individual consideration or feedback on our scale relevancy by a group of 10 gynecologists. The relevancy of the items was assessed using a four-point Likert scale: (1) not relevant, (2) somewhat relevant, (3) relevant, (4) very relevant. The recommended acceptable lower limit for CVI is 0.80 [19].

### Face validity

To test validity, to ensure the linguistic and conceptual equivalence of the translation, and to confirm the accuracy, appropriateness, and interpretation of the translated questionnaire, AR-PCOSQ was given to 30 patients with PCOS [20].

### Internal consistency

Internal consistency relates to a tool homogeneity. Cronbach's alpha coefficient value from 0 to 1 was utilized to evaluate the internal consistency. High value means that used tool is more reliable with low predictable errors. Cronbach's alpha coefficient value of ≥ 0.7 indicates acceptable internal consistency for the used tool [21].

### Test–retest reliability

Intraclass correlation-coefficient (ICC) value from 0 to 1 was utilized to evaluate the test–retest reliability that aims to determine the consistency of used tool when the same tool it is applied to the same participants at two different times. The following categories were selected to interpret the agreement levels: 00–0.2 as small, 0.21–0.40 as fair, 0.41–0.60 as moderate, 0.61–0.80 as substantial and 0.81–1 as almost perfect [22].

### Factor analysis

Factor analysis is a statistical tool to explain the AR-PCOSQ subscale factor structure variability. It is used to simplify the interpretation of factors and minimize the number of variables affected by each factor. The factor structure of the AR-PCOSQ was extracted by utilizing exploratory factor analysis (EFA) [23]. Kaiser–Mayer–Olkin (KMO) measure of adequacy in sampling and Bartlett’s test were used as well. KMO value greater than 0.6 indicates the sample is adequate [24, 25].

## Results

A total of 117 women with PCOS have been enrolled in the study. The mean age (years) and BMI (kg/m^2^) of subjects were 29.9 ± 6.33 and 27.21 ± 5.54, respectively. Most of the patients had ≥ 1 year long history of PCOS (73.5%) and a post-school degree (64.96%) (Table 1).

**Table 1:** Socio-demographic and clinical characteristic (n = 117)

Characteristics	
Age (years), mean ± SD	29.90 ± 6.33
BMI (kg/m^2^), mean ± SD	27.21 ± 5.54
*History of PCOS (years), n (%)*	
< 1 year	31 (26.5%)
≥ 1 year	86 (73.5%)
*Education level, n (%)*	
Pre-secondary school	3 (2.56%)
Secondary school	38 (32.48%)
Post-secondary school	76 (64.96%)

*SD* standard deviation

### Content validity

Ten gynecologists (4 males; and 6 females) whose ages range from 40 to 62 with at least 10 years of experience were contacted. The content validity index (CVI) for the AR-PCOSQ from 10 gynecologists was 0.9, indicating satisfactory validity content. The ten gynecologists rated almost all the translated items as relevant clinically and culturally with very few comments on some items that were slightly modified.

### Face validity

Thirty patients were interviewed for content validity. Their mean ages (years) and BMI (kg/m^2^) were 29 ± 7.08 and 26.7 ± 5.51, respectively. Most of the thirty patients had ≥ 1 year long history of PCOS (21 patients; 70%) and a post-school degree (17 patients; 56.7%). After the interview, it was ensured that the AR-PCOSQ was appropriate and relevant clinically and culturally, and that it was simple and understandable linguistically with no changes in wording required.

### Reliability

The internal consistency for reliability confirmation measured by Cronbach’s alpha coefficient was applied to all participants (n = 117). For each subscale and over all items, Cronbach's alpha Reliability coefficient was calculated. As shown in Table 2, all alpha coefficients (except that for menstrual problems) exceeded the recommended value of 0.70. Also, the alpha coefficients for all items together was 0.863, indicating good reliability. The intraclass correlation coefficients for each item for the 30 participants were also acceptable, ranging from 0.911 to 0.986 with *p* value < 0.001 (Table 3).

**Table 2:** Cronbach’s alpha of the AR-PCOSQ subscales and over all items (n = 117)

Subscales	Cronbach’s alpha	Mean	SD
Emotion and feelings	0.723	3.70	1.28
Body hair	0.888	4.01	2.08
Weight	0.849	3.65	1.88
Infertility	0.719	4.05	1.72
Menstrual problem	0.697	3.02	1.40
Over all items	0.863	3.67	1.08

*SD* standard deviation

**Table 3:** Intraclass correlation-coefficient (ICC) of the AR-PCOSQ subscales (n = 30)

Subscales	ICC	Mean test	SD test
		Mean re-test	SD re-test
Emotion and feelings	0.986*	23.67	10.08
		23.17	9.11
Body hair	0.986*	18.87	10.31
		18.37	9.43
Weight	0.951*	16.87	9.50
		16.60	8.06
Infertility	0.979*	16.90	7.56
		16.67	7.44
Menstrual problem	0.911*	12.70	7.79
		10.83	6.86

*SD* standard deviation

^*^p value < 0.001

### Factor analysis

The AR-PCOSQ was studied by conducting the key component analysis. The overall Kaiser–Meyer–Olkin sampling adequacy test was 0.772. The sphericity test of the Bartlett was significant (*p* ≤ 0.001) indicating that the variables correlated with one another. The AR-PCOSQ was found to have six factors. The number of explained variances ranged from 4.57 to 24.22%, with 64.44% of the overall variance explained (Table 4). Table 5 shows the factor loading from the AR-PCOSQ principal component analysis. The loading factor of the items is all greater than 0.514. In the last six items, different items were loaded in different factors.

**Table 4:** Total variance explained

Factor	% of variance
1	24.22
2	12.92
3	8.79
4	8.26
5	5.68
6	4.57
Total variance explained	64.44

**Table 5:** Factor loadings from the AR-PCOSQ principal component analysis

Q# and belonged subscale in the original questionnaire	Item	Factor					
		1	2	3	4	5	6
Q16: H	Embarrassment about excessive body hair	**0.889**	0.039	− 0.036	0.174	0.089	− 0.024
Q26: H	Growth of visible hair	**0.866**	0.030	− 0.077	0.184	0.158	0.013
Q15: H	Growth of visible hair on face	**0.857**	0.088	− 0.024	0.033	0.065	0.019
Q9: H	Growth of visible hair on upper lip	**0.748**	0.056	− 0.004	0.060	0.120	0.222
Q1: H	Growth of visible hair on chin	**0.691**	0.110	0.051	− 0.048	− 0.136	0.399
Q10: W	Trouble dealing with weight	0.171	**0.799**	0.010	0.068	− 0.053	0.117
Q3: W	Concern of being overweight	− 0.046	**0.791**	− 0.045	0.070	0.158	0.122
Q12: W	Felt frustration in trying to lose weight	− 0.009	**0.788**	0.073	0.245	0.020	0.030
Q24: W	Have difficulty staying at your ideal weight	0.167	**0.770**	0.165	0.000	0.100	0.133
Q22: W	Feel like not sexy as result of overweight	0.029	**0.641**	0.064	0.461	0.110	− 0.057
Q13: I	Felt afraid of not being able to have children	0.000	0.050	**0.907**	0.076	0.012	0.021
Q5: I	Concerned with infertility problems	− 0.092	0.009	**0.903**	0.077	0.012	0.034
Q25: I	Feel sad because of infertility problems	− 0.010	0.113	**0.824**	0.054	0.011	− 0.109
Q6: E	Moody as result of PCOS	0.124	0.183	0.046	**0.736**	0.127	0.079
Q2: E	Depressed for having PCOS	0.121	0.078	0.108	**0.704**	0.195	0.278
Q11: E	Low self-esteem as result of PCOS	− 0.006	0.189	0.193	**0.586**	− 0.251	− 0.017
Q4: E	Easily tired	0.309	0.084	− 0.110	**0.580**	0.456	0.016
Q7: M	Headaches	0.001	0.016	− 0.031	0.093	**0.695**	0.112
Q21: M	Menstrual cramps	0.132	0.054	− 0.115	0.100	**0.612**	0.148
Q19: M	Abdominal bloating	0.226	0.265	0.233	0.073	**0.551**	0.093
Q14: E	Felt frightened of getting cancer	− 0.049	0.047	0.338	0.019	**0.514**	0.092
Q18: E	Self-conscious as result of PCOS	0.039	0.221	0.045	0.249	− 0.032	**0.671**
Q20: M	Late menstrual period	0.266	0.095	− 0.126	− 0.049	0.267	**0.664**
Q8: M	Irregular menstrual problems	0.113	− 0.024	− 0.078	− 0.005	0.456	**0.598**
Q17: E	Worried about having PCOS	0.074	0.093	0.424	0.364	0.274	**0.544**
Q23: I	Feel lack of control over the situation with PCOS	0.102	0.122	− 0.006	0.511	0.271	**0.521**

The factor loadings of the items all exceed 0.514 (given in bold font)

*H* hirsutism, *W* weight, *I* infertility, *E* emotional and feelings, *M* menstrual

## Discussion

According to our findings of the PCOSQ in Arabic-speaking women, the Arabian PCOSQ version was culturally acceptable, applicable, and seemed relevant to their conditions. It has shown that the AR-PCOSQ version was overall relevant to all PCOS-related concerns as it expressed by our subjects. Despite of the fact that no item was recognized as missing, some items may be introduced or amended with further studies. In previous studies, acne domain was introduced as an essential domain to be involved into the PCOSQ as important factor to consider for assessing the wellbeing of women with PCOS [8, 17].

The satisfied internal consistency of our translated questionnaire (AR-PCOSQ) was obtained in almost all domains (body hair, emotions, weight, and infertility problems) as each domain scored above 0.7 on Cronbach's alpha correlation coefficient. However, the menstrual domain of the AR-PCOSQ scored 0.69 which is considered acceptable internal correlation coefficient according to George and Marley [26]. Similar studies among women from the United Kingdom, Canada, and Iran found similar results in achieving the reliability and validity in all domains with low value of correlation coefficient in the menstrual domains [8, 16, 18]. These low levels of achievement in the reliability and validity of the menstrual problem domain have been clarified differently. Guyatt et al. in Canada believes that the question regarding to headaches in the menstruation domain seemed not appropriate [18]. Bazarganipour et al. in Iran stated that relocating the question pointing to an irregular menstrual period (Question 8) from the emotional domain to the menstrual domain will significantly improved the reliability of both domains [17]. The menstrual domain in AR-PCOSQ will be assessed with more studies and utilization.

Test–retest reliability of the PCOSQ presented intra-class correlations above 0.9 in all the domains with *p* value < 0.001 (Table 3). That proved the stability and consistency of the PCOSQ over time. The period of two days to a week between the interviews was adopted to reduce recall bias while remaining within two weeks as stated by the questionnaires. Despite the fact that the second interview was done in another environment (at home through phone calls) while the initial assessment was in the obstetrics and gynecology clinics at KKUH, both were done by interviewing the participants to minimize the recall bias. Similar results of satisfactory intra-class correlation (ICC ranges from 0.71 to 0.92; *p* value > 0.05) were reported in the Iranian version of PCOSQ [27].

Different items of PCOSQ loaded in different factors were noticed when the original English version of PCOSQ was translated to other languages or performed in various societies [8, 12, 27]. Our factor analysis of the AR-PCOSQ revealed six factors: emotions and feelings, body hair, weight, infertility, menstrual problems, and new factor. Two factors in our study (weight and body hair) were similar to the original scale of PCOSQ, with the same items loading on each factor. The infertility factor was identical except for one item ("feel a lack of control over the situation with PCOS") that loaded on a new factor. One item ("felt frightened of getting cancer) and other two items ("self-conscious as result of PCOS", and "Worried about having PCOS”) of emotions and feeling factor were loaded on menstrual problems and the new factor, respectively. Two items ("late menstrual period" and "irregular menstrual problems") of menstrual problems factor were loaded into the new factor. These results are very similar to a study conducted by Jones et al. in the United Kingdom [8]. Therefore, moving some items from one factor to another may be considered. Bazarganipour et al. in the Iranian PCOSQ version moved the item "irregular menstrual problems" from the emotional factor to the menstrual factor, and the reliability of both factors improved significantly [27]. Therefore, we assume that classifying items such as "headaches", "menstrual cramps", "abdominal bloating”, and "felt frightened of getting cancer" under the psychosomatic characteristics factor, and moving the other unmatched item to menstrual problems may improve the reliability of our study; especially the reliability of the menstrual problems factor.

Although the adequate sample size was sufficient to enable factor analysis and test–retest reliability, several limitations of our study need to be mentioned. First, all the participants were from one medical center and one nationality (Saudi Arabia). Most of them had a high educational level, [64.96%, (Table 1)] and might have had good health literacy regarding their self-care and health-seeking behaviors. It is crucial for future researchers to validate the AR-PCOSQ using a sample from different regions and with subjects of various educational levels. Second, the responsiveness of the AR-PCOSQ was not assessed in this study due to the limited study period. Future research, along with sufficient follow-up time, should evaluate the ability of the AR-PCOSQ to describe the patient’s health status over time and its sensitivity to detect changes in HRQoL because of the intervention, and to determine the minimal clinical importance score to justify whether changes are clinically relevant.

## Conclusions

Our findings show the objective of establishing the initial reliability and validity of the Arabic version of the PCOSQ as a measure of the specific HRQoL in Saudi patients with PCOS. This will fill an important gap in measuring the HRQoL for patients with PCOS in research and community settings in Saudi Arabia. The PCOSQ is an excellent screening tool which health-care providers can be used to help prioritize health-related concerns from the patients’ perspective.

## Data Availability

The original (English) and translated (Arabic) polycystic ovarian syndrome questionnaire that support the findings of this study are available from McMaster University but restrictions apply to the availability of these questionnaires, which were used under license for the current study, and so are not publicly available. However, all data generated or analysed during this study are included in this published article.

## Abbreviations

AR-PCOSQ: Arabic version of the PCOSQ
BMI: Body mass index
CVI: Content validity index
EFA: Exploratory factor analysis
HRQoL: Health-related quality of life
ICC: Intraclass correlation-coefficient
IRB: Institutional review board
KKUH: King Khalid University Hospital
KOM: Kaiser–Mayer–Oklin
PCOS: Polycystic ovarian syndrome
PCOSQ: Polycystic ovarian syndrome questionnaire
